# A Secreted Glutamate-Specific Serine Protease from *Staphylococcus aureus* Activates NF-κB Signaling and Enhances Vascular Leakage

**DOI:** 10.3390/pathogens15090915

**Published:** 2026-08-31

**Authors:** Jong Woo Park, Jung Eun Park

**Affiliations:** 1 Department of Agricultural Biology, National Institute of Agricultural Sciences, Wanju-gun 55365, Jeonbuk-do, Republic of Korea; jwpark0824@korea.kr; 2 BK21 FOUR Educational Research Group for Age-Associated Disorder Control Technology, Department of Integrative Biological Science, Chosun University, Jeonnam-Gwangju 61452, Republic of Korea

**Keywords:** *Staphylococcus aureus*, VSPase, glutamate-specific serine protease, NF-κB, inflammatory mediators, vascular permeability, virulence-associated protease

## Abstract

*Staphylococcus aureus* secretes multiple proteases that contribute to tissue damage and modulation of host immune responses. The glutamate-specific serine endopeptidase (VSPase) is secreted by *S. aureus* strain C-66; however, its effects on inflammatory signaling and vascular permeability remain poorly understood. In this study, we investigated the pro-inflammatory and vascular permeability-enhancing activities of VSPase and compared them with those of its catalytically inactive S237L mutant. In RAW 264.7 macrophages, VSPase induced the mRNA expression of TNF-α, IL-1β, IL-12β, MIP-2, cyclooxygenase-2, and prostaglandin E synthase. VSPase also increased TNF-α secretion in a concentration-dependent manner, whereas S237L elicited little or no comparable response. VSPase caused a rapid and transient reduction in cytosolic IκBα levels and increased NF-κB DNA-binding activity in nuclear extracts. Competition and supershift assays confirmed the specificity of the DNA–protein complex and demonstrated the presence of the p65 subunit. In a guinea pig Evans blue extravasation assay, intradermal administration of VSPase increased vascular leakage in a dose-related manner, whereas S237L produced minimal effects. Collectively, these findings demonstrate that VSPase induces pro-inflammatory mediator expression in association with activation of a p65-containing NF-κB complex and enhances vascular permeability in vivo. The markedly reduced activity of S237L further suggests that the catalytic activity of VSPase is closely associated with its inflammatory and vascular effects. VSPase may therefore contribute to host inflammatory responses and vascular dysfunction during staphylococcal infection.

## 1. Introduction

*Staphylococcus aureus* is an important Gram-positive pathogen that causes diseases ranging from localized skin and soft tissue infections to pneumonia, bacteremia, sepsis, infective endocarditis, and toxic shock syndrome [1,2,3]. Its pathogenicity is mediated by numerous surface-associated and secreted virulence factors that promote colonization, immune evasion, tissue damage, and bacterial dissemination [4,5]. Among these factors, extracellular proteases can alter the host environment by cleaving structural proteins, immune mediators, and components of epithelial or vascular barriers. The major extracellular proteases produced by *S. aureus* include the metalloprotease aureolysin, the glutamate-specific serine protease V8 protease/SspA, and the cysteine proteases staphopain A/ScpA and staphopain B/SspB [6,7]. However, the inflammatory and vascular effects of individual staphylococcal proteases remain incompletely defined.

Macrophages are important components of the innate immune response to *S. aureus* infection and produce inflammatory cytokines, chemokines, and lipid mediators in response to bacterial factors [8,9]. Many of these responses are regulated by nuclear factor-κB (NF-κB). In resting cells, NF-κB proteins are retained in the cytoplasm through their association with inhibitor of κB proteins [10]. Following stimulation, a reduction in IκBα levels permits NF-κB activation and the expression of inflammatory mediators, including tumor necrosis factor-α (TNF-α), interleukin-1β (IL-1β), chemokines, cyclooxygenase-2 (COX-2), and prostaglandin biosynthesis-related enzymes [11,12]. Excessive production of these mediators may contribute to local tissue inflammation and systemic complications during bacterial infection.

Increased vascular permeability is another characteristic of acute inflammation and severe bacterial infection [13,14]. Staphylococcal proteases may enhance vascular leakage through direct cleavage of host barrier components or through the production of vasoactive mediators [15]. Staphopain A has been shown to induce bradykinin B_2_ receptor-dependent vascular leakage, whereas staphopain B had no detectable vascular leakage activity by itself but augmented the effect of staphopain A [16]. A previous report also noted that purified aureolysin and V8 protease did not directly increase dermal vascular permeability under the conditions examined, although the supporting data were not presented in detail [16]. Other studies have demonstrated that V8 protease can cleave numerous host proteins and compromise epithelial barrier integrity, suggesting that its effects may depend on the host tissue and experimental context [15,17]. Whether highly homologous V8-like proteases from different *S. aureus* strains differ in their inflammatory or vascular activities therefore remains unclear.

We previously purified and characterized VSPase, a glutamate-specific serine endopeptidase secreted by *S. aureus* strain C-66 [18]. Sequence comparison indicated that VSPase shares approximately 97% amino acid identity with V8 protease/SspA, identifying it as a highly homologous member of the staphylococcal glutamyl endopeptidase family. Site-directed mutagenesis identified His119, Asp161, and Ser237 as residues required for detectable proteolytic activity, and substitution of Ser237 with leucine generated the catalytically inactive S237L mutant [18]. In the present study, we examined whether VSPase induces pro-inflammatory mediator expression and NF-κB activation in RAW 264.7 macrophages. We compared VSPase with S237L to evaluate the association between catalytic activity and the observed cellular responses and investigated whether VSPase enhances vascular permeability in vivo using an Evans blue extravasation assay.

## 2. Materials and Methods

### 2.1. Materials

Dulbecco’s modified Eagle’s medium (DMEM) and fetal bovine serum (FBS) were obtained from BioWhittaker (Walkersville, MD, USA). Lipopolysaccharide (LPS; Sigma-Aldrich, St. Louis, MO, USA), histamine, Evans blue, and formamide were purchased from Sigma-Aldrich (St. Louis, MO, USA). The mouse TNF-α enzyme-linked immunosorbent assay (ELISA) kit was obtained from R&D Systems (Minneapolis, MN, USA). Polyvinylidene difluoride (PVDF) membranes were purchased from Bio-Rad Laboratories (Richmond, CA, USA). Antibodies against IκBα and glyceraldehyde-3-phosphate dehydrogenase (GAPDH), as well as the appropriate horseradish peroxidase-conjugated secondary antibodies, were obtained from Santa Cruz Biotechnology (Santa Cruz, CA, USA). The anti-NF-κB p65 antibody used for the supershift assay was purchased from Cell Signaling (Boston, MA, USA). Protein molecular weight markers were obtained from Fermentas (Darmstadt, Germany). Unless otherwise indicated, all other reagents were of analytical grade.

### 2.2. Cell Culture

RAW 264.7 (American Type Culture Collection, Manassas, VA, USA) murine macrophages were obtained from ATCC (Manassas, VA, USA) and cultured in DMEM supplemented with 10% FBS and 1% penicillin–streptomycin at 37 °C in a humidified atmosphere containing 5% CO_2_. For each experiment, cells were treated with LPS, VSPase, or S237L at the concentrations and for the periods indicated below.

### 2.3. Preparation of VSPase and S237L

VSPase and its catalytically inactive S237L mutant were cloned, expressed, and pu-rified as previously described [18]. Protein purity and proteolytic activity were assessed as described previously [18], and representative data are presented in Figure S1. To assess potential endotoxin contamination, endotoxin levels in the purified VSPase and S237L preparations were quantified using the ToxinSensor™ Chromogenic LAL Endotoxin Assay Kit (GenScript, Piscataway, NJ, USA) according to the manufacturer’s instructions. Endotoxin levels in both VSPase and S237L preparations were below the detection limit of the assay (<0.005 EU/mL). Purified proteins were stored at −20 °C until use.

### 2.4. RNA Extraction and Reverse Transcription-Polymerase Chain Reaction (RT-PCR)

RAW 264.7 cells were treated with LPS (1 μg/mL), VSPase (3 μg/mL), or S237L (3 μg/mL) for the indicated periods. Untreated cells were used as controls. Following treatment, total RNA was extracted using the easy-spin Total RNA Extraction Kit (iNtRON Biotechnology, Seongnam, Republic of Korea) according to the manufacturer’s instructions.

Complementary DNA was synthesized from 1 μg of total RNA using an oligo(dT)_18_ primer and M-MLV reverse transcriptase (Bioneer, Daejeon, Republic of Korea). PCR amplification was performed using the resulting cDNA in reaction mixtures containing 2.5 mM dNTPs, 2.5 U of Taq DNA polymerase, and 10 pmol each of the forward and reverse primers listed in Table S1. The primer sequences used in this study were previously reported by Park [19], and detailed information on the target genes, gene functions, and primer sources is provided in Table S1. The PCR buffer contained 1.5 mM MgCl_2_, 50 mM KCl, and 10 mM Tris-HCl (pH 8.3).

Amplification was performed using a thermal cycler (Eppendorf, Hamburg, Germany) under the following conditions: denaturation at 94 °C for 30 s, annealing at the primer-specific temperatures listed in Table S1 for 30 s, and extension at 72 °C for 40 s, for a total of 45 cycles. The amplified products were separated by electrophoresis on 2% agarose gels, stained with ethidium bromide, and visualized under ultraviolet illumination. GAPDH was used as an internal control. Band intensities from three independent RT-PCR experiments were quantified by densitometric analysis, normalized to GAPDH, and expressed relative to the corresponding 0-h value using ImageJ software 1.41 (National Institutes of Health, Bethesda, MD, USA).

### 2.5. Enzyme-Linked Immunosorbent Assay (ELISA)

**Figure 1:**
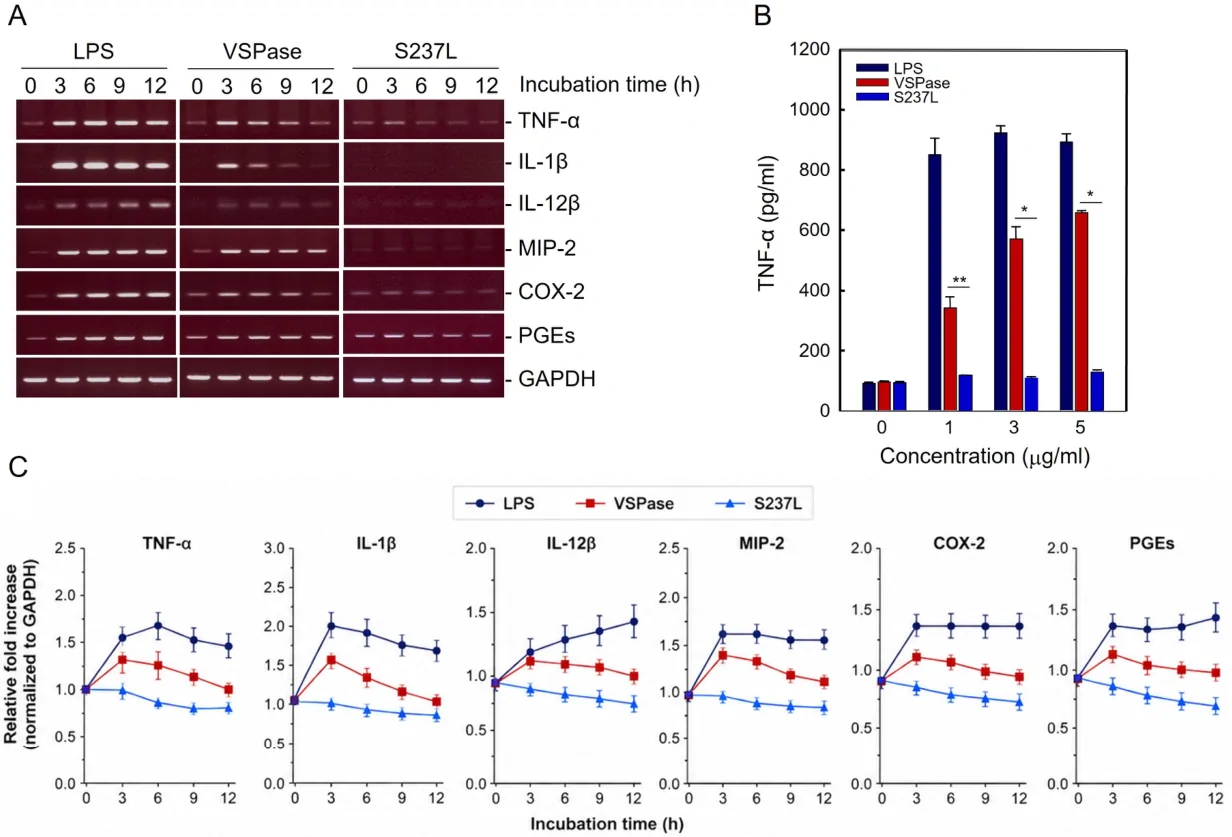
VSPase induces pro-inflammatory mediator expression and TNF-α secretion in RAW 264.7 macrophages. (**A**) RAW 264.7 cells were treated with LPS (1 μg/mL), VSPase (3 μg/mL), or S237L (3 μg/mL) for the indicated periods. The mRNA expression levels of TNF-α, IL-1β, IL-12β, MIP-2, COX-2, and PGES were examined by RT-PCR. GAPDH was used as an internal control. The results shown are representative of three independent experiments. (**B**) RAW 264.7 cells were treated with the indicated concentrations of LPS, VSPase, or S237L for 3 h, and TNF-α concentrations in the culture supernatants were determined by ELISA. Data are presented as the mean ± SD of three independent experiments. Statistical differences were analyzed using two-way ANOVA followed by Šidák multiple-comparison test. * *p* < 0.05, ** *p* < 0.01 for comparisons between VSPase and S237L at the corresponding concentrations. (**C**) Densitometric analysis of the RT-PCR products shown in (**A**). Band intensities were normalized to GAPDH, and the normalized value at 0 h was set to 1.0. Data are expressed as relative fold changes and presented as the mean ± SD of three independent experiments.

RAW 264.7 cells were seeded in 48-well plates at a density of 5 × 10^5^ cells/well and cultured for 24 h before stimulation. Cells were treated with the indicated concentrations of LPS, VSPase, or S237L for 3 h at 37 °C. Based on Figure 1B, the treatment concentrations were 0, 1, 3, and 5 μg/mL.

Following treatment, culture supernatants were collected and clarified by centrifugation at 13,000× *g* for 10 min. TNF-α concentrations in the supernatants were measured using a mouse TNF-α ELISA kit (R&D Systems, Minneapolis, MN, USA) according to the manufacturer’s instructions. Samples were analyzed in triplicate, and TNF-α concentrations were calculated from a standard curve.

### 2.6. SDS-PAGE and Western Blotting

RAW 264.7 cells were treated with LPS (1 μg/mL) or VSPase (3 μg/mL) for the indicated periods. Following treatment, the cells were washed with ice-cold PBS and lysed in ice-cold RIPA buffer. The lysates were centrifuged at 13,000× *g* for 10 min at 4 °C. The resulting supernatants were collected as whole cell lysates.

Protein concentrations were determined using the Bradford assay. Equal amounts of whole-cell protein were mixed with SDS–PAGE sample buffer, heated at 100 °C for 3 min, and separated by SDS–PAGE. Proteins were transferred onto PVDF membranes, which were blocked for 2 h at room temperature with Tris-buffered saline containing 0.1% Tween 20 and 5% skim milk.

The membranes were incubated overnight at 4 °C with antibodies against IκBα or GAPDH diluted in blocking buffer. After three washes with TBS-T, the membranes were incubated with the appropriate horseradish peroxidase-conjugated secondary antibodies for 2 h at room temperature. The membranes were subsequently washed three times with TBS-T and once with TBS. Immunoreactive bands were detected using WestZol Western Blotting Detection Reagent (iNtRON Biotechnology, Seongnam, Republic of Korea), and chemiluminescent signals were captured on X-ray film. GAPDH was used as a loading control.

### 2.7. Nuclear Extracts Preparation and Electrophoretic Mobility Shift Assay (EMSA)

Nuclear extracts were prepared from RAW 264.7 cells before and after stimulation with VSPase for the indicated time periods. Cell pellets were resuspended in 100 µL of ice-cold hypotonic buffer containing 10 mM HEPES (pH 7.9), 0.5 mM KCl, 1.5 mM MgCl_2_, 0.5 mM dithiothreitol (DTT), and 0.2 mM phenylmethylsulfonyl fluoride (PMSF). After incubation on ice for 5 min, the samples were centrifuged at 15,000× *g* for 5 min. The resulting nuclear pellets were resuspended in extraction buffer containing 20 mM HEPES (pH 7.9), 25% glycerol, 1.5 mM MgCl_2_, 0.8 M KCl, 0.2 mM ethylenediaminetetraacetic acid (EDTA), 0.5 mM DTT, and 0.2 mM PMSF and incubated for 20 min at 4 °C. The samples were subsequently centrifuged at 15,000× *g* for 20 min at 4 °C, and the supernatants containing the nuclear proteins were collected and aliquoted. Protein concentrations were determined using the Bradford assay reagent (Sigma-Aldrich, St. Louis, MO, USA), and equal amounts of nuclear protein were used for electrophoretic mobility shift assays (EMSAs).

A double-stranded oligonucleotide containing the NF-κB consensus-binding sequence (5′-AGCTTGGGGACTTTCC-3′) was end-labeled using 5 U of T4 polynucleotide kinase and 50 µCi of [γ-^32^P]ATP (3000 Ci/mmol; Amersham Pharmacia Biotech, Uppsala, Sweden). Binding reactions were performed in a final volume of 20 µL containing 10 µg of nuclear extract, 0.5 pmol of radiolabeled probe, 20 mM HEPES (pH 7.9), 80 mM NaCl, 0.1 mM EDTA, 1 mM DTT, 5% glycerol, and 250 ng of poly(dI-dC). Protein–DNA complexes were resolved on 6% non-denaturing polyacrylamide gels in 0.25× TBE buffer containing 22.2 mM Tris, 22.2 mM boric acid, and 0.5 mM EDTA. The gels were then subjected to autoradiography using X-ray film, as previously described [20]. For supershift assays, nuclear extracts were incubated with an anti-p65 antibody before the addition of the radiolabeled probe, as described previously [21].

### 2.8. Vascular Permeability Assay

Four-week-old male Hartley guinea pigs weighing 250–350 g were purchased from Damul Science (Daejeon, Republic of Korea) and housed under standard laboratory conditions with controlled temperature and humidity, a 12-h light/dark cycle, and free access to food and water. The animals were anesthetized with diethyl ether (Junsei Chemical Co., Tokyo, Japan). Evans blue dye was administered intravenously via the marginal ear vein at a dose of 65 mg/kg body weight, followed by intradermal injection of 50 µL of each test sample prepared in 10 mM phosphate-buffered saline (PBS) into separate sites on the shaved flank skin. Before administration, the Evans blue solution (5% in 0.6% saline) was passed through a 0.2 µm membrane filter. Ten minutes after intradermal injection, the animals were euthanized by exsanguination under anesthesia. The blue-stained skin tissue surrounding each injection site was excised and incubated in 3 mL of formamide at 60 °C for 48 h to extract the extravasated dye. The amount of Evans blue extracted from each tissue sample was determined by measuring the absorbance at 620 nm and expressed as micrograms of extravasated Evans blue. Each treatment was administered to a separate intradermal site within the same animal. Three guinea pigs were used, and each animal received all treatments; therefore, the individual animal was considered the experimental unit (*n* = 3 animals).

## 3. Results

### 3.1. VSPase Induces Pro-Inflammatory Mediator Expression in Raw 264.7 Cells

VSPase and its catalytically inactive mutant, S237L, were prepared as previously described [18]. RAW 264.7 cells were treated with LPS, VSPase, or S237L for the indicated periods, and the mRNA expression of pro-inflammatory cytokines, chemokines, and prostaglandin biosynthesis-related enzymes was assessed by RT-PCR.

VSPase markedly induced the expression of TNF-α, IL-1β, IL-12β, and MIP-2, whereas S237L elicited little or no detectable induction of these genes (Figure 1A). TNF-α, IL-1β, and MIP-2 expression increased rapidly following VSPase treatment, indicating an early inflammatory response. In comparison, IL-12β induction appeared at later time points. VSPase also increased the expression of COX-2 and prostaglandin E synthase (PGES). COX-2 expression was induced predominantly during the early phase of treatment, whereas PGES expression increased more gradually. In contrast, S237L had minimal effects on the expression of either enzyme. Semi-quantitative densitometric analysis of three independent experiments showed a similar pattern after normalization to GAPDH (Figure 1C). Consistent with these transcriptional changes, VSPase stimulated TNF-α secretion in a concentration-dependent manner (Figure 1B). TNF-α levels increased to approximately 657 pg/mL following treatment with 5 μg/mL VSPase, whereas TNF-α production in S237L-treated cells remained near basal levels across the concentrations examined. Taken together, these results demonstrate that VSPase activates multiple pro-inflammatory responses in RAW 264.7 macrophages and support the involvement of its proteolytic activity in this process.

### 3.2. VSPase Activates NF-κB Signaling in RAW 264.7 Cells

**Figure 2:**
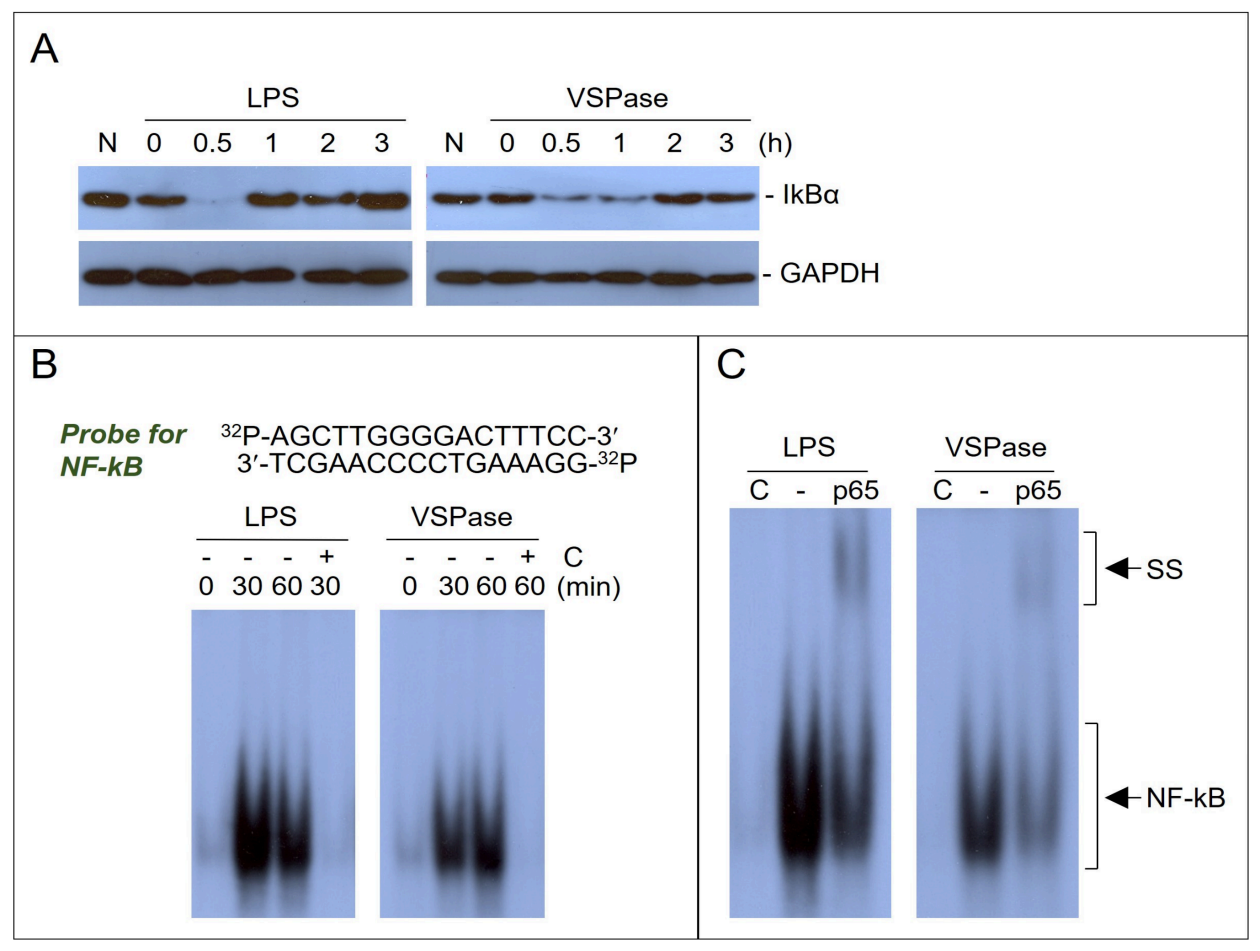
VSPase induces IκBα degradation and activates NF-κB DNA-binding activity in RAW 264.7 macrophages. (**A**) RAW 264.7 cells were treated with LPS (1 μg/mL) or VSPase (3 μg/mL) for the indicated periods. Whole-cell lysates were prepared, and IκBα protein levels were examined by immunoblotting. GAPDH was used as a loading control. (**B**) Nuclear extracts prepared from RAW 264.7 cells treated with LPS or VSPase for the indicated periods were incubated with a ^32^P-labeled consensus NF-κB oligonucleotide probe, and NF-κB DNA-binding activity was analyzed by electrophoretic mobility shift assay (EMSA). For competition assays, an excess of unlabeled NF-κB consensus oligonucleotide was added to the binding reaction (+). (**C**) The composition of the NF-κB DNA–protein complex was examined by supershift assay. Nuclear extracts from LPS- or VSPase-treated cells were incubated with the radiolabeled NF-κB probe in the absence (−) or presence of an anti-p65 antibody (p65). Addition of the anti-p65 antibody generated a supershifted complex (SS), confirming the presence of the p65 subunit in the NF-κB complex. C, competition assay with excess unlabeled NF-κB probe; N, untreated control. The results shown are representative of three independent experiments.

The induction of pro-inflammatory mediators by VSPase prompted us to examine whether VSPase activates the NF-κB signaling pathway. Immunoblot analysis of whole-cell lysates showed that IκBα levels were markedly and transiently reduced within 30 min of VSPase treatment and subsequently recovered at later time points (Figure 2A). A similar transient reduction in IκBα was observed following LPS stimulation.

NF-κB DNA-binding activity was subsequently examined by electrophoretic mobility shift assay using nuclear extracts from stimulated RAW 264.7 cells. VSPase treatment increased the formation of an NF-κB-specific DNA–protein complex at 30 and 60 min, as also observed in LPS-treated cells (Figure 2B). The shifted complex was markedly reduced by the addition of excess unlabeled NF-κB consensus oligonucleotide, supporting the specificity of the DNA-binding reaction.

To determine whether the VSPase-induced NF-κB complex contained the p65 subunit, a supershift assay was performed using an anti-p65 antibody. Addition of the anti-p65 antibody generated a more slowly migrating supershifted complex and reduced the mobility of the original NF-κB complex in both LPS- and VSPase-treated samples (Figure 2C). These results demonstrate that VSPase induces transient IκBα degradation and increases the nuclear DNA-binding activity of a p65-containing NF-κB complex in RAW 264.7 macrophages.

### 3.3. VSPase Induces Vascular Leakage

**Figure 3:**
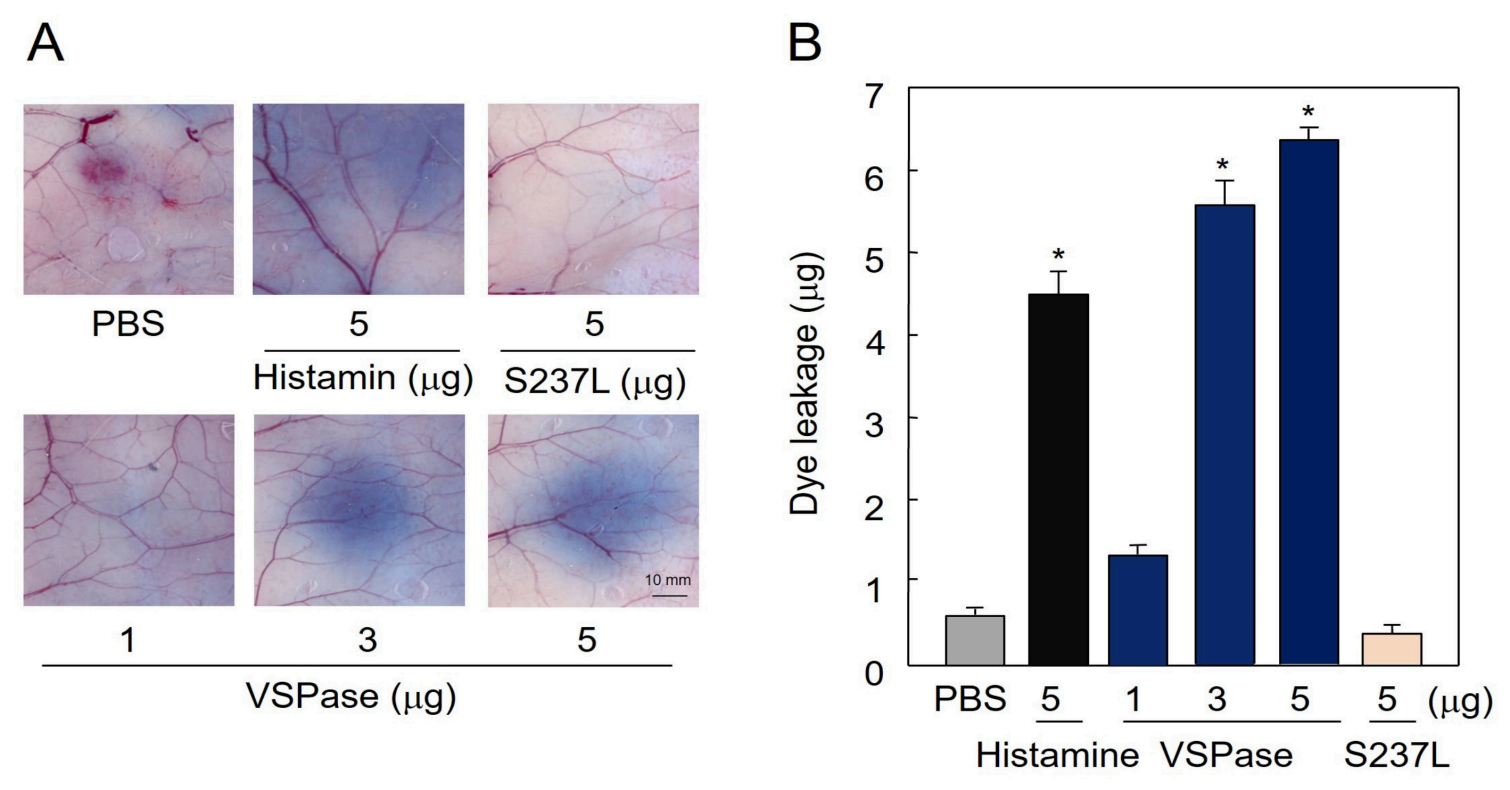
VSPase induces vascular leakage in vivo. (**A**) Representative images of Evans blue extravasation following intradermal injection of PBS, histamine (5 μg), VSPase (1, 3, or 5 μg), or the catalytically inactive S237L mutant (5 μg). Histamine was used as a positive control. Each treatment was administered to a separate intradermal site within the same animal. (**B**) Evans blue extracted from the injection sites was quantified as a measure of vascular leakage. Data are presented as the mean ± SD of three animals (*n* = 3). Statistical significance was analyzed using repeated-measures one-way ANOVA followed by Dunnett’s multiple-comparisons test, with each treatment compared with the PBS-treated site. * *p* < 0.005 versus PBS.

The effect of VSPase on vascular permeability was examined using an Evans blue extravasation assay. Intradermal injection of VSPase produced visible dye extravasation that increased with the administered dose (Figure 3A). VSPase at 1 μg induced a modest increase in dye leakage, whereas 3 and 5 μg VSPase elicited marked vascular leakage. Histamine, used as a positive control, also induced substantial dye extravasation. In contrast, the catalytically inactive S237L mutant at 5 μg produced little or no detectable vascular leakage.

Quantitative analysis confirmed that VSPase increased Evans blue extravasation in a dose-related manner, reaching approximately 6.4 μg at a VSPase dose of 5 μg (Figure 3B). By comparison, dye leakage following treatment with 5 μg S237L remained comparable to that observed in the PBS-treated group. These findings demonstrate that VSPase enhances vascular permeability in vivo and indicate that this activity is closely associated with its proteolytic function.

## 4. Discussion

In the present study, we investigated the contribution of VSPase, a glutamate-specific serine endopeptidase secreted by *Staphylococcus aureus* strain C-66, to host inflammatory responses and vascular permeability [18]. VSPase induced the expression of pro-inflammatory cytokines, chemokines, and prostaglandin biosynthesis-related enzymes in RAW 264.7 macrophages, increased NF-κB DNA-binding activity, and promoted vascular leakage in vivo (Figure 1, Figure 2 and Figure 3). In contrast, the catalytically inactive S237L mutant elicited little or no comparable response. These findings support a close association between the proteolytic activity of VSPase and its inflammatory and vascular permeability-enhancing effects, although they do not exclude the possible contribution of other structural or functional properties of the enzyme.

VSPase increased the mRNA expression of TNF-α, IL-1β, IL-12p40, and MIP-2, whereas S237L induced minimal or undetectable responses (Figure 1A). Consistent with these transcriptional changes, VSPase also increased TNF-α secretion in a concentration-dependent manner, whereas S237L had little effect (Figure 1B). TNF-α and IL-1β are major mediators of acute inflammation that promote leukocyte activation and endothelial responses during bacterial infection [22,23]. MIP-2 is a murine neutrophil-recruiting chemokine that contributes to inflammatory cell accumulation at sites of infection [24,25]. Thus, the induction of these mediators suggests that VSPase may amplify macrophage-mediated inflammatory responses during staphylococcal infection.

VSPase also increased the mRNA expression of COX-2 and prostaglandin E synthase (PGES) during the early phase of treatment (Figure 1A). COX-2 and PGES are key enzymes involved in prostaglandin biosynthesis and are commonly induced during inflammatory responses [26,27]. However, because prostaglandin production was not directly measured, the present results do not establish that VSPase increases prostaglandin synthesis. Rather, the observed transcriptional changes suggest that VSPase may engage prostaglandin-associated inflammatory pathways.

Activation of NF-κB may contribute to the inflammatory response induced by VSPase. VSPase caused a rapid and transient reduction in IκBα levels, which was evident within 30 min and followed by recovery at later time points (Figure 2A). This response is consistent with activation of the canonical NF-κB pathway [11,12]. EMSA further demonstrated increased NF-κB DNA-binding activity in nuclear extracts from VSPase-treated cells (Figure 2B). The specificity of the DNA–protein complex was supported by competition with an excess of unlabeled NF-κB probe, and the supershift assay using an anti-p65 antibody confirmed the presence of the p65 subunit in the complex (Figure 2C). These findings support the activation of a p65-containing NF-κB complex by VSPase. However, because NF-κB inhibition, knockdown, or reporter assays were not performed, the extent to which NF-κB is required for VSPase-induced inflammatory gene expression remains to be determined. The upstream receptor or proteolytic substrate responsible for initiating this response also remains unknown.

The in vivo vascular permeability assay showed that VSPase increased Evans blue extravasation in a dose-related manner, whereas S237L produced little or no detectable effect (Figure 3). These findings further support an association between VSPase catalytic activity and its vascular permeability-enhancing effect. One possible mechanism is the direct proteolytic cleavage of endothelial junctional proteins, basement membrane components, or extracellular matrix proteins involved in maintaining vascular integrity [28,29]. Alternatively, cytokines and other inflammatory mediators induced by VSPase may indirectly enhance vascular permeability through endothelial activation or the release of vasoactive mediators from mast cells and other host cells [30,31]. Because endothelial barrier disruption, mast cell activation, and vasoactive mediator release were not directly examined, the present data do not distinguish between these potential mechanisms.

This study has several limitations. The inflammatory effects of VSPase were evaluated primarily in the murine RAW 264.7 macrophage cell line, and confirmation using primary macrophages, human immune cells, and vascular endothelial cells is required. In addition, the vascular permeability experiment was performed using a relatively small number of animals (*n* = 3), which represents a limitation of the present study. Furthermore, tissue weights were not recorded; therefore, Evans blue extravasation could not be normalized to tissue weight, which represents an additional limitation of this study. Although the results obtained with S237L support the involvement of catalytic activity, additional evidence from protease inhibitors, independent catalytic mutants, and substrate-identification studies would strengthen this conclusion. Functional inhibition or genetic suppression of NF-κB will also be necessary to determine whether NF-κB activation is required for VSPase-induced inflammatory mediator expression. Finally, infection experiments using isogenic VSPase-deficient and complemented *S. aureus* strains will be required to establish the contribution of VSPase to inflammation, vascular dysfunction, and bacterial virulence in vivo.

**Figure 4:**
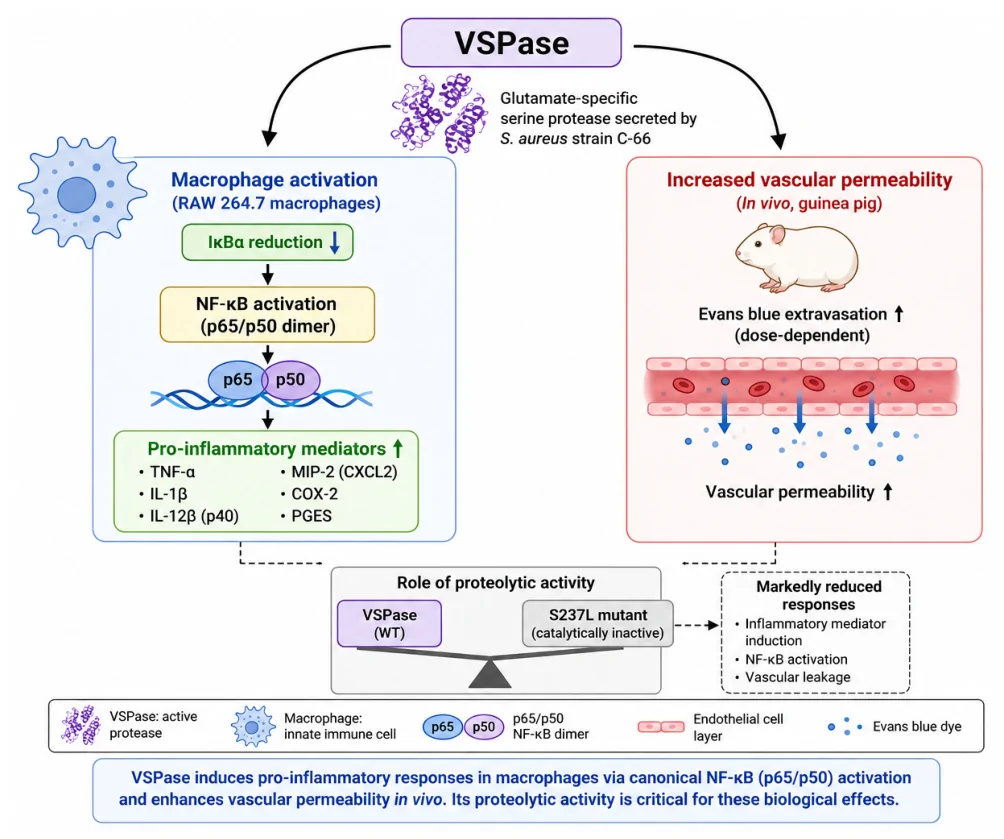
Schematic summary of the biological effects of VSPase. VSPase induces pro-inflammatory mediator expression and NF-κB activation in RAW 264.7 macrophages and enhances vascular permeability in vivo. The markedly reduced responses induced by the catalytically inactive S237L mutant suggest that these biological effects are closely associated with the proteolytic activity of VSPase.

From a practical perspective, these findings may provide a basis for exploring VSPase as a potential anti-virulence target in *S. aureus* infection. Because the catalytically inactive S237L mutant showed markedly reduced inflammatory and vascular effects, inhibition of VSPase proteolytic activity may represent a potential strategy for limiting protease-mediated host tissue injury and vascular dysfunction. However, further studies using specific VSPase inhibitors and relevant infection models are required to evaluate this possibility. A schematic summary of the inflammatory and vascular effects of VSPase and the proposed role of its proteolytic activity is presented in Figure 4.

## 5. Conclusions

In conclusion, VSPase induced the expression of multiple pro-inflammatory mediators, including TNF-α, IL-1β, IL-12β, MIP-2, COX-2, and PGES, and increased TNF-α secretion in RAW 264.7 macrophages. VSPase also promoted IκBα loss and increased the DNA-binding activity of a p65-containing NF-κB complex. In vivo, VSPase enhanced vascular permeability in a dose-related manner, whereas the catalytically inactive S237L mutant produced markedly reduced responses. These findings provide new evidence linking the proteolytic activity of VSPase to inflammatory signaling and vascular permeability. Collectively, our results suggest that VSPase may function as an extracellular virulence-associated factor contributing to inflammatory and vascular responses during *S. aureus* infection. Further identification of its host substrates and evaluation in bacterial infection models will be important for defining its contribution to staphylococcal pathogenesis and its potential as a target for anti-virulence strategies.

## Data Availability

The data presented in this study are available on request from the corresponding author.

## Supplementary Materials

The following supporting information can be downloaded at: https://www.mdpi.com/article/10.3390/pathogens15090915/s1, Figure S1: Construction, purification, and enzymatic validation of recombinant VSPase and the S237L mutant. (A) Schematic representation of the domain organization of the 336-amino-acid VSPase precursor, including the N-terminal signal peptide, propeptide, and mature enzyme region. The catalytic triad residues His119, Asp161, and Ser237 are indicated. (B) Schematic map of the pFLAG-ATS expression vector carrying the VSPase or S237L coding sequence between the *Hind*III and *Kpn*I restriction sites. The vector contains an N-terminal OmpA signal sequence and FLAG tag, the *tac* promoter, *lacI*, the f1 origin of replication, and an ampicillin-resistance gene. (C) SDS-PAGE analysis of purified VSPase and S237L. The VSPase and S237L lanes were adapted from our previous study [18]. Non-adjacent lanes from the same gel were juxtaposed for presentation. (D) Relative proteolytic activities of purified VSPase and S237L. The activity of wild-type VSPase was defined as 100%. Data are presented as the mean ± SD of three independent experiments; Table S1: Gene information and primer sequences used for RT-PCR analysis.

## Abbreviations

The following abbreviations are used in this manuscript:

ANOVA	*analysis of variance*
ATP	adenosine triphosphate
cDNA	complementary DNA
COX-2	cyclooxygenase-2
DMEM	Dulbecco’s modified Eagle’s medium
DTT	dithiothreitol
EDTA	ethylenediaminetetraacetic acid
ELISA	enzyme-linked immunosorbent assay
EMSA	electrophoretic mobility shift assay
FBS	fetal bovine serum
GAPDH	glyceraldehyde-3-phosphate dehydrogenase
HEPES	4-(2-hydroxyethyl)-1-piperazineethanesulfonic acid
IκBα	inhibitor of κB alpha
IL-1β	interleukin-1 beta
IL-12β	interleukin-12 beta
LPS	lipopolysaccharide
MIP-2	macrophage inflammatory protein-2
M-MLV	Moloney murine leukemia virus
mRNA	messenger RNA
NF-κB	nuclear factor kappa B
PBS	phosphate-buffered saline
PGES	prostaglandin E synthase
PMSF	phenylmethylsulfonyl fluoride
PVDF	polyvinylidene difluoride
RIPA	radioimmunoprecipitation assay
RT-PCR	reverse transcription polymerase chain reaction
SD	standard deviation
SDS–PAGE	sodium dodecyl sulfate–polyacrylamide gel electrophoresis
TBE	Tris–borate–EDTA
TBS	Tris-buffered saline
TBS-T	Tris-buffered saline containing Tween 20
TNF-α	tumor necrosis factor alpha
VSPase	glutamate-specific serine protease from *Staphylococcus aureus* strain C-66
