# Supplementary material for: A Secreted Glutamate-Specific Serine Protease from Staphylococcus aureus Activates NF-κB Signaling and Enhances Vascular Leakage
Source: Pathogens. 2026 Aug 31;15(9):915. doi: 10.3390/pathogens15090915 (PMC13610798; doi:10.3390/pathogens15090915)
Supplement: Supplementary file 1 [file pathogens-15-00915-s001.zip › Supplementary Figure S1 .pdf]

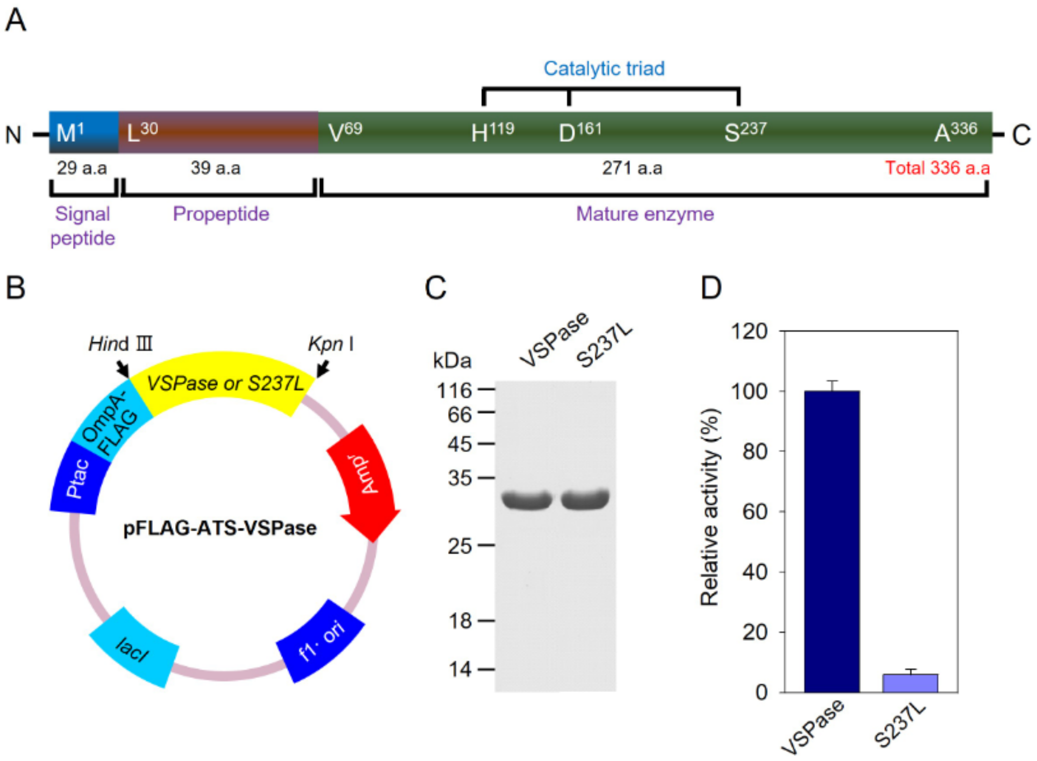

Supplementary Figure S1. Construction, purification, and enzymatic validation of recombinant VSPase and the S237L mutant. (A) Schematic representation of the domain organization of the 336-amino-acid VSPase precursor, including the N-terminal signal peptide, propeptide, and mature enzyme region. The catalytic triad residues His119, Asp161, and Ser237 are indicated. **(B)** Schematic map of the pFLAG-ATS expression vector carrying the VSPase or S237L coding sequence between the *Hind*III and *Kpn*I restriction sites. The vector contains an N-terminal OmpA signal sequence and FLAG tag, the *tac* promoter, *lacI*, the f1 origin of replication, and an ampicillin-resistance gene. **(C)** SDS-PAGE analysis of purified VSPase and S237L. The VSPase and S237L lanes were adapted from our previous study [18]. Non-adjacent lanes from the same gel were juxtaposed for presentation. **(D)** Relative proteolytic activities of purified VSPase and S237L. The activity of wild-type VSPase was defined as 100%. Data are presented as the mean ± SD of three independent experiments.
