# Supplementary material for: A Secreted Glutamate-Specific Serine Protease from Staphylococcus aureus Activates NF-κB Signaling and Enhances Vascular Leakage
Source: Pathogens. 2026 Aug 31;15(9):915. doi: 10.3390/pathogens15090915 (PMC13610798; doi:10.3390/pathogens15090915)
Supplement: Supplementary file 1 [file pathogens-15-00915-s001.zip › Supplementary Table S1.pdf]

Supplementary Table S1. Gene information and primer sequences used for RT-PCR analysis.

Target	Gene name and function	Primer	Primer sequence (5' to 3')	Expected size (bp)	Annealing temperature (℃)	GenBank Accession No.
TNF-α	Tumor necrosis factor alpha; pro-inflammatory cytokine	Forward	TTTCCTCCCAATACCCCTTC	197	48	Y00467
		Reverse	AGTGCAAAGGCTCCAAAGAA			
IL-1β	Interleukin-1 beta; pro-inflammatory cytokine	Forward	TGTGAAATGCCACCTTTTGA	205	50	AK168047
		Reverse	GTAGCTGCCACAGCTTCTCC			
IL-12β	Interleukin-12 beta subunit (p40); cytokine subunit involved in cell-mediated immune responses	Forward	GGGACATCATCAAACCAGAC	226	53	NM_008352
		Reverse	ACTTCGGGTAGATGTCTTCTC			
Cox-2	Cyclooxygenase-2; inducible enzyme involved in prostaglandin biosynthesis	Forward	CAGCAAATCCTTGCTGTTCC	658	50	BC052900
		Reverse	CCATCCTTGAAAAGGCGCAG			
PGEs	Prostaglandin E synthase; enzyme involved in PGE_2 biosynthesis	Forward	ATGCCTTCCCCGGGCCTG	462	48	AK150013
		Reverse	TCACAGATGGTGGGCCAC			
MIP-2	Macrophage inflammatory protein-2 (CXCL2); neutrophil-recruiting chemokine	Forward	AACAAAGGCAAGGCTAACTGA	203	53	X53798
		Reverse	AACATAACAACATCTGGGCAAT			
GAPDH	Glyceraldehyde-3-phosphate dehydrogenase; internal control gene	Forward	TCAGCAATGCATCCTGCACCAC	252	55	BC082592
		Reverse	TGCCAGTGAGCTTCCCGTTCAG			

Primer sequences were previously used in the study by Park (2008)[19]. F, forward primer; R, reverse primer.
